# Knowledge support for ankle fractures in the Swedish Fracture Register – a qualitative study of physicians’ experiences

**DOI:** 10.1186/s12913-022-07799-5

**Published:** 2022-03-23

**Authors:** Emilia Möller Rydberg, Johan Insulan, Ola Rolfson, Maziar Mohaddes, Linda Ahlstrom

**Affiliations:** 1 Institute of Clinical Sciences, Sahlgrenska Academy, University of Gothenburg, Gothenburg, Sweden; 2 Department of Orthopaedics, Sahlgrenska University Hospital, Region Västra Götaland, Göteborgsvägen 31, SE-431 80 Mölndal, Gothenburg, Sweden; 3 Institute of Health and Care Sciences, Sahlgrenska Academy, University of Gothenburg, Gothenburg, Sweden

**Keywords:** Knowledge support, Swedish Fracture Register, Ankle Fracture, Semi structured interviews

## Abstract

**Background:**

The aim of this study is to investigate the experiences of physicians presented with a knowledge support system while registering data on ankle fractures in the Swedish Fracture Register. The present study aims to answer the following research questions:

• “How is receiving knowledge support while registering a fracture in the Swedish Fracture Register experienced by the physicians using it?”.

• “Can a feeling of increased usability of a quality register be achieved by providing the user with real-time feedback?”.

**Methods:**

A total of 20 physicians using the Swedish Fracture Register were recruited using a purposive sampling strategy. Qualitative content analysis was performed on individual semi-structured interviews performed in May and June 2020.

**Results:**

The present study demonstrates that the knowledge support system in the Swedish Fracture Register was perceived by the physicians as strengthening the evidence base and improving the quality of ankle fracture treatment. The knowledge support system was evaluated as a good tool for validating clinical decisions and managing the information that needs to be processed to make informed decisions.

**Conclusions:**

The present study affirms that being provided with knowledge support is appreciated by physicians, increase value for work and enhance the initiative to register. The physicians experienced that the knowledge support provided an appreciated validation of the clinical decisions taken and a feeling of improved care. When incorporating knowledge support into an NQR, consideration must be given to physicians’ fears of becoming overly reliant on a template and losing control of the clinical base.

## Background

The complex information environment in healthcare constitutes a challenge for physicians [1]. Making informed decisions is cumbersome, as the amount of information that needs to be processed is constantly increasing [2]. There are numerous examples of ways in which these challenges could be mitigated through computer-aided systems [3]. The first attempts to recruit computers to aid in diagnosis and treatment decisions were made almost 50 years ago [4, 5]. Despite this, there are as yet only a few clinical decision support systems (CDSS) in use [6]. CDSSs are systems designed to provide physicians with clinical decision support, i.e. assistance in clinical decision-making by providing patient-specific recommendations. Another computer-aided support in clinical decision-making is knowledge bases or knowledge support systems (KSS). These systems provide knowledge, but, unlike the CDSS, they do not have a link to electronic medical records (EMR) or patient-specific data. KSSs do not give patient-specific recommendations or aid in decision-making but merely present the clinician with knowledge that already exists.

The Swedish Fracture Register (SFR) is a national quality register that prospectively collects data on all types of fracture [7]. Ankle fractures have been registered in the SFR since 2012 and classified into 14 different fracture groups and subgroups according to the AO/OTA classification system [8]. The SFR has been gradually adopted since 2010 and, since 2020, all the orthopedic departments in Sweden participate in registering fractures in the SFR. The process of entering data in the SFR is a web-based, three-step procedure performed by the physician at patient presentation at the accident and emergency department (A&E), described in detail by Wennergren et al. [7]. Both non-surgically treated and surgically treated fractures are registered. Several studies have been conducted and they reveal substantial accuracy and high reliability in terms of fracture classification in the SFR for several injury locations [9–11]. Ankle fractures are the third most common type of fracture, treated daily at orthopedic departments across the world [12].

In 2020, a knowledge support system (KSS) was introduced in the SFR for eight different AO/OTA groups of ankle fractures. The aim of the KSS was to improve the care of patients with ankle fractures, increase the incentive for the individual physician to register ankle fractures in the SFR and broaden the use of the SFR.

Since the use of CDSSs and KSSs is still limited in national quality registers (NQR) in Sweden, knowledge of the way physicians perceive this feedback in their daily work is scarce. Receiving knowledge support and feedback is probably experienced differently from person to person. The experiences most likely play an important role in the outcome of introducing new KSSs and the impact on the subsequent care of patients. Knowledge of how physicians experience receiving feedback on their management of fracture patients could improve future KSSs and aid in ensuring evidence-based care for orthopedic patients.

The aim of the present study is to investigate physicians’ experiences of being presented with a knowledge support system while registering data in the SFR.

The present study aims to answer the following research questions:

- “How is receiving knowledge support while registering a fracture in the Swedish Fracture Register experienced by the physicians using it?”
- “Can the usability of a quality register and the feeling of providing high-quality care be increased by giving the user real-time feedback?”

## Methods

Four orthopedic departments in Sweden (Göteborg, Karlstad, Falun and Gävle) were approached about their interest in participating in a pilot project testing the KSS for three months. All four departments agreed to participate in the project and the KSS was launched in the SFR at the participating departments on 25 February 2020. The KSS was designed as a three-step model, following the steps in the data-entering process performed by the physician in the SFR. Step one ensured that the chosen classification was correct. Step two presented information on the recommended treatment method for the classified fracture group or subgroup. This step also presented data from the register on how this fracture group had been treated over the past year at the physician’s own department and in Sweden as a whole. Step three posed the question of whether or not the recommended treatment was chosen and if not why not. Information on recommended treatment method was derived from a structured evidence-based treatment algorithm for ankle fracture management used at Sahlgrenska University hospital. During the pilot project, the KSS was active for three months, 25 February-25 May 2020. The physicians at the four participating orthopedic departments were exposed to the KSS when registering any of the selected eight groups of ankle fractures. During the three months, a total of 98 physicians came in contact with the KSS, registering a total of 200 ankle fractures. At the end of the pilot project, 20 semi-structured interviews were performed to capture the physicians’ experiences of the KSS. The interviews were analyzed using qualitative content analysis (QCA) [13, 14].

### Design

The present study is a qualitative interview study analyzed using QCA according to Graneheim and Lundman [13, 15]. Conventional QCA was performed with an inductive approach, looking for similarities and differences in the interviews. The Standards for Reporting on Qualitative Research (SRQR) described by O’Brien were followed to improve transparency [16].

### Study population

Purposive sampling [17] was used to capture a variety of knowledge and experience within the phenomena of interest. On 28 April 2020, the heads of the four participating departments were approached by email asking for physicians willing to participate in the interviews. Consultant surgeons, associate specialists, residents, interns and junior doctors with a range of different ages, sex and experience of working in an orthopedic department were requested from the four participating departments. Five physicians from each of the four departments volunteered, making up the study group of 20 interviewees. Thirteen of the interviewees were male and 7 female and their experience of working in an orthopedic department ranged from a few months to over 20 years (Table 1).

**Table 1:** Demographic data of the study/interview participants with experience of working in an orthopedic department

Participant	Sex	Age group, yrs	Position	Range of experience, yrs	Hospital, n
P1	Male	30 – 40	Resident	5 – 10	1
P2	Female	30 – 40	Orthopedic surgeon	5 – 10	1
P3	Male	50 – 60	Consultant	> 20	1
P4	Male	30 – 40	Resident	< 5	1
P5	Female	20 – 30	Intern	< 1	2
P6	Male	20 – 30	Junior doctor	< 1	3
P7	Male	40 – 50	Orthopedic surgeon	10 – 20	2
P8	Male	50 – 60	Consultant	10 – 20	3
P9	Male	40 – 50	Orthopedic surgeon	5 – 10	4
P10	Female	30 – 40	Resident	5 – 10	4
P11	Male	20 – 30	Intern	< 1	2
P12	Male	30 – 40	Orthopedic surgeon	10 – 20	4
P13	Male	40 – 50	Orthopedic surgeon	5 – 10	2
P14	Female	30 – 40	Resident	< 5	2
P15	Female	30 – 40	Orthopedic surgeon	5 – 10	1
P16	Female	20 – 30	Resident	< 5	4
P17	Male	40 – 50	Orthopedic surgeon	5 – 10	3
P18	Male	20 – 30	Junior doctor	< 1	3
P19	Female	20 – 30	Junior doctor	< 1	3
P20	Male	50 – 60	Consultant	> 20	4

### Data collection

In May and June 2020, 20 semi-structured qualitative research interviews were conducted [14], all by the same interviewer (EMR). EMR works at one of the participating orthopedic departments and has developed the KSS in the SFR. To reduce the risk of this influencing the data collection a semi-structured interview guide was constructed by the whole research team (EMR, JI, LA). JI is a medical student without previous experience in orthopedics or of the KSS in the SFR. LA is a nurse with experience of qualitative research but no prior experience of the SFR or the KSS studied. All the interviews started with demographic questions and an initial open question regarding experiences of using the KSS. The interview guide, used as needed, covered the following areas: experience of obtaining feedback regarding choice of treatment, contributions by the KSS to daily work, influence on decisions relating to classification and treatment by the KSS, suggestions for further improvements and emotional response to using the KSS. The interviews were conducted in Swedish, in person (5 interviews) or via the digital network Zoom (15 interviews), between May and June 2020. The recorded part of the interviews lasted a mean of 8 min (median 8.5 min), the initial demographic questions were answered before recording started. All the interviews were recorded, anonymized and transcribed by a third party. Moreover, the interviewees were given an identification number, used for the statements in the results section (Table 1).

### Data analysis

The data analysis and coding was performed by three individual researchers (EMR, JI and LA). The transcribed interviews were read through by the interviewer (EMR) and two other researchers (JI, LA) and corrected for missing words and transcription mistakes. Data were analyzed using QCA according to Graneheim and Lundman [13]. Computer-assisted qualitative data analysis software, Nvivo 12 (QSR International), was used to aid in data organization. First, the twenty interviews were read through by three individual researchers (EMR, JI, LA) to acquire a sense of the whole. Parts containing meaningful information were then identified and extracted as meaning units (MU). The MUs were then condensed and abstracted into short descriptive sentences which were labeled with a code. The codes were compared based on differences and similarities and grouped in sub-categories and further in categories (Table 2). The categories were then analyzed and interpreted with regard to latent content and grouped into four themes (Table 3). Discrepancies in interpretation were discussed and re-examined by the researchers until consensus was reached; to ensure consistency in the application of categories, additional researchers were involved (OR, MM). OR and MMR are experienced orthopedic surgeons but have limited experience of the SFR and the KSS studied. The quotes, MUs, codes, sub-categories, categories and themes were translated from Swedish to English by a professional third-party translator.

**Table 2:** Example of how the analysis was performed

Meaning unit (MU)	Condensed MU	Code	Sub-category	Category
*“If I hadn’t had that pop-up message, I would have prescribed five to six weeks in plaster, but it recommended four weeks with an orthosis, which I thought was suitable and so I did that”*	Would have prescribed plaster but changed to orthosis and shortened immobilization time	The function has changed action	The effect the function has on decisions	Action

**Table 3:** Sub-categories, categories and themes relating to the KSS in the SFR

Sub-category	Category	Theme
Validate decisions relating to action	Validation	Enhancing quality control of the decisions made
Food for thought		
Support for decisions relating to action	Action	
The effect the function has on decisions		
Stop thinking for yourself	The physician	Being afraid of losing control
Increased workload		
Human factor		
The function is blunt	The patient	
Basis of evidence		
Thoughts on extensions of the function	Suggestions	Acknowledging the benefits associated with a KSS
Thoughts on improvements of the function		
Positive thoughts about the function	Experiences	
Wide range of use for the SFR		
Positive experiences in relation to receiving feedback		
Lack of information	Implementation	Managing the organizational obstacles in healthcare
Experience of the function	Organization	
Overall organization		

### Ethics

Regarding the interviewees, a great deal of consideration was paid to preserving anonymity among the physicians who were interviewed. All the interviews were anonymized and informed consent was obtained prior to the interviews. Regarding the data in the SFR, no data were extracted from the SFR for this study and, as a result, no specific ethical considerations had to be made in this respect. The study was approved by the Swedish Ethical Review Authority (ref no 2020–00,867).

## Results

Four main themes were identified while investigating the experiences of physicians presented with a KSS while registering data in the SFR. The themes were: “Enhancing the quality control of the decisions made”, “Being afraid of losing control”, “Acknowledging the benefits associated with a KSS” and “Managing the organizational obstacles in healthcare” (Table 3).

### Theme: Enhancing the quality control of the decisions made

The first theme, “Enhancing the quality control of the decisions made”, contains conceptions relating to the impact of the KSS on the decisions made by the individual physician. The theme consists of two categories: “Validation” and “Action”, both related to effects of the KSS on validating planned decisions and the actions taken by the physician after being presented with the KSS.

#### Category: Validation

The first category, “Validation”, covers sub-categories related to the validation of decisions made and food for thought for the physician when in contact with the KSS. The statement demonstrates that the encounter with the KSS spurred consideration of whether or not the decision that was taken was the correct one.

“I mean, it really doesn’t matter how experienced you are. You can sometimes think in the wrong way and make overly hasty decisions and then you are given an extra reminder.” – P13.

The interviewees believed that the KSS improved the decisions that were taken by providing a reminder to the physician of the recommended treatment.

“I can imagine that, if you treat fractures in a way that is clearly incorrect, you repeatedly hear ‘you don’t normally do this’ or ‘no one else does this’. In this case, I think it would lead you to think again.” – P12.

“You have to think again if you deviate too much from the recommendations and what is regarded as… standard treatment, but you have to think it through and justify any deviations.” – P7.

#### Category: Action

The second category, “Action”, contains conceptions about who benefits from the KSS and the actions the KSS has led to for the physician. One common perception was that inexperienced physicians would benefit most from a KSS. “If you don’t have that much experience, you perhaps think again and maybe even change your mind. I think it is of the greatest value to younger doctors.” – P15.

For some of the physicians, the KSS had clearly impacted their decisions. For others, the statements indicate that this might have been the case had the physician been exposed to the KSS more often or in situations where the right decision was not so evident to them. “If I hadn’t had that pop-up message, I would have prescribed five to six weeks in plaster, but it recommended four weeks with an orthosis, which I thought was suitable and so I did that.” – P9.

### Theme: Being afraid of losing control

The second theme, “Being afraid of losing control”, contains conceptions related to the risk of the KSS obstructing thinking for yourself and the risk of the KSS being too blunt with respect to the patient. The theme contains two categories: “The physician” and “The patient”.

#### Category: The physician

Being “told” by a computer-aided system which treatment is recommended was experienced as something troublesome by some of the physicians. Many of them thought that this was specifically worrying for the most inexperienced physicians. The statements showed that there was a fear of becoming overly dependent on a template and stopping thinking for yourself. “Then it’s extremely important that it isn’t written in stone, so that people understand that it’s a recommendation and doesn’t apply to everyone. There are very specific cases in which we don’t operate.” – P15. “It’s important to strike a balance between when it is too much and when we stop thinking for ourselves.” – P2.

#### Category: The patient

Despite the KSS being based a on recent and in-depth evidence base, there was a worry among the physicians about relying too much on it, since the information is not specific to the individual patient in front of you. “I think the approach differs when the patients’ age differs. The geriatric sub-population is very special because the complication frequency is fairly high, so you may need other recommendations.” – P8.

### Theme: Acknowledging the benefits associated with a KSS

The third theme, “Acknowledging the benefits associated with a KSS”, consists of two categories related to experiences of coming in contact with the KSS and suggestions for further development. The categories are: “Suggestions” and “Experiences”.

#### Category: Suggestions

Most of the physicians had thoughts on and suggestions for further improvements and expansions of the KSS. The statements demonstrated large-scale interest in the subject of the broader use of computer-aided systems in healthcare to ensure more evidence-based decisions. “If the feedback is good, it would be useful to have it for more common fractures.” – P15. “If the window only appears when you actually deviate.” – P13.

#### Category: Experiences

Despite the risk mentioned of a KSS making physicians overly dependent on a template, the statements in the category of “Experiences” indicate a widespread perception that the KSS enhanced the quality of care provided and extended the range of use for the SFR. “Yes, I think it’s good. It enhances the quality. I think it also acts as a support in terms of decision-making. Great!” – P3. “What does the literature actually say and what do you do because you simply do things? I think it’s good to be given a reminder.” – P10.

The statements also demonstrate that the KSS provided an incentive for the physician to register ankle fractures in the SFR, one of the aims when introducing the KSS. “I would think about it next time I register… or if I come across an injury like this, I would register it immediately and I would see the help and support that was available.” – P7.

### Theme: Managing the organizational obstacles in healthcare

The final theme, “Managing the organizational obstacles in healthcare”, covers statements related to a lack of information about the introduction of the KSS and the organizational obstacles to a smooth strategic flow in healthcare. The theme contains two categories: “Implementation” and “Organization”.

#### Category: Implementation

Despite detailed information via email before the pilot project started, some physicians did not know about the KSS or had not noticed the KSS in the SFR on their first encounter. This could be interpreted as the KSS not disturbing the workflow but being found to be well integrated in the daily work. “Yes, I really didn’t understand why it appeared. But then I thought OK, but it’s just… a little information. So, yes.” – P17.

Some physicians had been surprised by the information provided by the KSS, making them reflect on the way ankle fractures are managed and the basis for decisions taken in the department and in Sweden as a whole. “…but then you think, what the hell, we have a memo that’s very clear. Have we failed to follow it in 35% of cases?” – P4.

#### Category: Organization

As fractures are not always registered to the SFR immediately in the A&E, the physician might not encounter the KSS until after a decision has already been made. Some regarded this as a problem and others reflected on the fact that they would remember the information they had been given when the next patient presented, thereby still benefiting from the KSS. “I have probably encountered it more times than I realized. After I started to think about it, I have met two patients for whom I could have used the function.” – P19.

## Discussion

The present study demonstrates that the KSS in the SFR was perceived by the physicians registering fractures in the SFR as strengthening the evidence base and improving the quality of ankle fracture treatment.

To our knowledge, no previous study has been conducted of the way clinicians perceive receiving knowledge support while using a national quality register in their clinical everyday work. This study will provide a better understanding of the way clinicians perceive being exposed to knowledge support. This could be useful for all NQRs planning to expand their area of use in order to be of greater value to the users. This study also adds to the understanding surrounding the reasoning of orthopedic surgeons when deviating from recommended treatment, information that could be of use when planning future efforts to enhance the evidence base in medicine.

One way of aiding clinicians in making evidence-based decisions in healthcare is by introducing structured guidelines or treatment algorithms [18]. This was found to be successful by both Wykes et al. and Jain et al., who implemented evidence-based guidelines for ankle fracture treatment [19, 20]. They showed that the number of radiographs, days immobilized and days without permitted weight-bearing were reduced significantly, saving both economic resources and patient discomfort, without increasing complications [19]. The KSS studied is derived from the evidence-based treatment algorithm for ankle fractures used at Sahlgrenska University hospital. By incorporating the treatment algorithm into the SFR the information was made more easy available for physicians in Sweden and equivalent information was present for more ankle fracture patients, enhancing the prerequisites for equal care.

Recent studies have shown that computer-aided systems in healthcare need to be well integrated in electronic systems that are already in use but not in electronic health records, to minimize extra workload and minimize alert fatigue [3, 21]. Systems that require a reason for over-riding the advice provided have shown a high success rate [21, 22]. The present study investigated a KSS in an NQR that is used every day at all the orthopedic departments in Sweden, the SFR. The final step of the KSS posed a question about whether the recommended treatment was followed, asking the physician to explain the reason for over-riding the recommendation. As the statements demonstrate, the KSS was perceived as “a natural and fairly short extension of what we already do” and was found to improve the decisions taken by physicians.

Studies have reported on the effectiveness of clinical knowledge support systems in improving care and reducing diagnostic errors [2, 23]. A remarkable error reduction was found among physicians in Japan who were exposed to a computer-based system compared with those not exposed to the system [2]. In the US, the same electronic clinical knowledge support system was found to be associated with improved health outcomes [23]. The aim of the KSS studied here was to improve the care of patients with ankle fractures, an aim that was realized according to the statements from the interviewees. Evaluating if patient related outcome measures or complication rates were affected by the introduction of the KSS are outside the scope of this article but remain an important topic for future studies.

Our assessment is that the present study has a high level of trustworthiness. The fact that the interviewees had various experiences and a variation in both gender and age adds to the credibility, which is further reinforced by demonstrations in tables and text of how meaning units were condensed and abstracted. As far as the method of choice is concerned, our assessment is that QCA is the most appropriate method for the research questions posed and QCA has the advantage of investigating both the manifest content and the latent interpretation of what is said. In our opinion, using QCA in an area like this, where the theories and literature are scarce, is a good method for exploring the experiences of physicians. A further argument in favor of a high level of trustworthiness in this study is the fact that the overall data collection was conducted over a short period of time and all the interviews were conducted by the same interviewer (EMR), providing high dependability. The transferability of our results beyond Sweden may be limited, as no other country has a national fracture register like the SFR. However, we believe that the results could be transferred to other NQRs and health systems like the Swedish ones.

One limitation of this paper is that it coincided with the Covid-19 pandemic, resulting in fewer registered ankle fractures at the participating departments than expected and, as a result, less experience among the physicians coming in contact with the KSS [24]. The pandemic also resulted in all the interviews outside SU being conducted via Zoom, possibly missing some aspects of communication between the interviewer and the interviewees. We still argue that there is large-scale transferability in this study, since it contains so many aspects of experience and the interviewees were recruited from four different hospitals. Interviewees with a variety in age, sex and of experiences working in an orthopedic department volunteered and were interviewed, but in the nature of voluntary selection lies that the physicians volunteering might be more positive, or more negative, to the KSS and hence wanting to express their opinions. The fact that the interviewer (EMR) works at one of the departments enrolled in the study and has been a part in developing the KSS in the SFR might have affected the interviewees to share more positive experiences. However, 15 of the interviewees were from three other departments and did not have information on EMRs affiliation or prior engagement in the KSS. The rest of the research-team (JI, LA) have no previous experience with the KSS in the SFR and JI did not work at any orthopedic department at the time of the study.

There is widespread agreement that knowledge support systems and decision support increase the quality of healthcare for both patients and doctors. In spite of this, only a few knowledge support systems are in use. The present study demonstrates that clinicians appreciate being presented with knowledge support while using a national quality register in their clinical work. Further studies are needed to explore how KSSs can be effectively incorporated into NQRs, thereby increasing the value for physicians and patients.

## Conclusions

The present study affirms that being provided with knowledge support is appreciated by physicians, increase value for work and enhance the initiative to register. The physicians experienced that the knowledge support provided an appreciated validation of the clinical decisions taken and a feeling of improved care. When incorporating knowledge support into an NQR, consideration must be paid to the fears of physicians of becoming overly reliant on a template and losing control of the clinical base.

## Data Availability

The datasets used and analyzed during the current study are not publicly available due to the large amount of text material but are avaliable from the corresponding author on reasonable request.

## Abbreviations

NQR: National Quality Register
SFR: Swedish Fracture Register
CDSS: Clinical decision support systems
KSS: Knowledge support systems
EMR: Electronic medical records
AO/OTA: Arbeitsgemeinschaft für Osteosynthesefragen/Orthopaedic Trauma Association
A&E: Accident and emergency department
QCA: Qualitative content analysis
SRQR: The Standards for Reporting on Qualitative Research
MU: Meaning units
